# Estimation of the absorbed dose in simultaneous digital breast tomosynthesis and mechanical imaging

**DOI:** 10.1117/1.JMI.12.S1.S13003

**Published:** 2024-07-24

**Authors:** Anna Bjerkén, Hanna Tomic, Sophia Zackrisson, Magnus Dustler, Predrag R. Bakic, Anders Tingberg

**Affiliations:** a Lund University, Department of Translational Medicine, Faculty of Medicine, Medical Radiation Physics, Malmö, Sweden; b Skåne University Hospital, Department of Hematology, Oncology and Radiation Physics, Radiation Physics, Malmö, Sweden; c Lund University, Department of Translational Medicine, Faculty of Medicine, Diagnostic Radiology, Malmö, Sweden

**Keywords:** digital breast tomosynthesis, mechanical imaging, digital breast tomosynthesis mechanical imaging, radiation dose measurement

## Abstract

**Purpose:**

Use of mechanical imaging (MI) as complementary to digital mammography (DM), or in simultaneous digital breast tomosynthesis (DBT) and MI – DBTMI, has demonstrated the potential to increase the specificity of breast cancer screening and reduce unnecessary biopsies compared with DM. The aim of this study is to investigate the increase in the radiation dose due to the presence of an MI sensor during simultaneous image acquisition when automatic exposure control is used.

**Approach:**

A radiation dose study was conducted on clinically available breast imaging systems with and without an MI sensor present. Our estimations were based on three approaches. In the first approach, exposure values were compared in paired clinical DBT and DBTMI acquisitions in 97 women. In the second approach polymethyl methacrylate (PMMA) phantoms of various thicknesses were used, and the average glandular dose (AGD) values were compared. Finally, a rectangular PMMA phantom with a 45 mm thickness was used, and the AGD values were estimated based on air kerma measurements with an electronic dosemeter.

**Results:**

The relative increase in exposure estimated from digital imaging and communications in medicine headers when using an MI sensor in clinical DBTMI was 11.9%±10.4. For the phantom measurements of various thicknesses of PMMA, the relative increases in the AGD for DM and DBT measurements were, on average, 10.7%±3.1 and 11.4%±3.0, respectively. The relative increase in the AGD using the electronic dosemeter was 11.2%±<0.001 in DM and 12.2%±<0.001 in DBT. The average difference in dose between the methods was 11.5%±3.3.

**Conclusions:**

Our measurements suggest that the use of simultaneous breast radiography and MI increases the AGD by an average of 11.5%±3.3. The increase in dose is within the acceptable values for mammography screening recommended by European guidelines.

## 1 Introduction

Screening for early breast cancer detection has been shown to reduce breast cancer mortality. The current screening standard in Europe is digital mammography (DM). The limitations of DM screening are related to sensitivity (by missed cancers) and specificity (by false positives), particularly in women with high breast density.^1^^–^^5^ Digital breast tomosynthesis (DBT), a pseudotomographic breast imaging method, is a newer approach that utilizes an acquisition geometry similar to that of DM. The X-ray tube moves around the breast in an arc, acquiring multiple low-dose projection images with a total dose level similar to that of DM.^6^^,^^7^ Compared with DM, reconstructed DBT slices contain less overlapping normal tissue that can mask existing tumors or cause false positives.

Although European guidelines recommend DBT as an alternative to DM in screening, there remain concerns regarding the elevated incidence of false-positive findings associated with DBT.^8^^,^^9^ DBT increases the visibility of tissue details, which helps with the detection of more cancers but also increases false-positive findings. This has been noticed in European DM screening programs, which have a relatively low recall rate compared with those in the US.^6^^,^^10^^–^^12^ However, in other settings, the false positives and recalls have been shown to be reduced with DBT.^13^^–^^15^ It has also been shown that negative psychosocial consequences—namely, anxiety—follow false-positive screening results among women who receive invitations for a clinical workup.^16^^,^^17^

One potential approach to address this issue could be to use mechanical imaging (MI) of the breast as an adjunct to radiographic imaging.^18^ MI is a form of elasticity imaging in which stress patterns over the surface of the compressed breast can be measured. These MI findings are comparable to palpation of the breast, except that MI provides quantitative measurements of the relative stiffness of the breast interior. Promisingly, the integration of MI has demonstrated the potential to enhance specificity in breast cancer screening.^19^^–^^21^ Dustler et al. observed a noteworthy 36% reduction in false positives when MI was incorporated alongside DM (DMMI) in two separate acquisitions.^20^ Our institution has developed a multimodality approach named simultaneous DBT and MI (DBTMI) that aims to utilize the combined advantages of DBT and MI to augment both sensitivity and specificity while preserving the clinical workflow.^22^^–^^26^ Preliminary study of clinical DBTMI suggested an improvement in specificity, comparable to DMMI.^24^

However, the presence of an MI sensor during simultaneous DBT and MI acquisition raises concerns over potential increases in the radiation dose. Factors such as breast tissue thickness and composition and other materials within the X-ray beam can influence automatic exposure control (AEC), which sets acquisition parameters to maintain the requisite image quality. The specific composition of the MI sensor used in our study, containing metallic (silver) elements within a plastic covering, has not been publicly disclosed. In a prior investigation, we employed simulations in which we approximated the sensor composition at 75% plastic and 25% silver by comparing the contrast between sensor elements and plastic in both simulated and clinical images.^25^ This particular issue concerning the dose in DBTMI is not present in the case of DMMI, for which the workflow is different. In our DMMI studies, recalled women are invited to participate in the MI study. MI is performed by acquiring a low-dose mammogram with exposure values of 5 mAs (corresponding to an additional dose to the women at about 5% of the traditional DM). Then, the MI image is matched with the corresponding diagnostic or screening mammogram. In DBTMI, the acquisition can be performed simultaneously because artifacts can be reduced in the clinical image through post-processing based on flat-fielding.^22^^,^^27^ However, the exposure settings need to be adapted to maintain consistent image quality, which affects the dose.

Previously, we conducted a preliminary dose comparison between DBTMI and conventional DBT in 20 women who had been recalled from DM screening due to suspicious findings. That study relied on estimated doses retrieved from digital imaging and communications in medicine (DICOM) headers and suggested that the presence of an MI sensor increased exposure by approximately 10%.^28^ In this study, we aim to expand the dose comparison between clinical DBT and DBTMI to confirm our preliminary results. Several factors could have affected our previous results—mainly the small sample size but also the potential variation in breast thickness arising from breast repositioning. To address this, we analyze the dose with and without an MI sensor in clinical image acquisitions and using solid, uncompressible polymethyl methacrylate (PMMA) blocks routinely used for quality control assessments.

This study aims to comprehensively assess the dose increment in terms of average glandular dose (AGD) resulting from the presence of the MI sensor in DBT mode during simultaneous acquisition. The observed dose increase in DBTMI is balanced by the improved cost efficiency of breast cancer screening, in terms of reduced diagnostic workup (with a potential 30% to 40% reduction in false positive diagnoses and unnecessary biopsies),^24^ reduced psychological burden to women, and enhanced clinical workload.

## 2 Materials and Methods

This study estimates the increase in AGD when using simultaneous MI and breast radiography. All measurements were conducted using clinical breast radiography systems available at Unilabs AB and Skåne University Hospital, Malmö, Sweden. Our estimations were based on three approaches. In the first approach, clinical data were analyzed by comparing exposure values from clinical acquisitions of DBT with acquisitions of DBTMI in the same woman. Using DICOM header information enabled a fast and convenient comparison of the exposure values. As previously mentioned, corresponding clinical analysis was not possible in the DM case because the workflow in DMMI is different than that of DBTMI. In our second approach, phantom measurements were analyzed by comparing AGD values from each mammographic system with and without the MI sensor present and repeated for various phantom thicknesses in both DM and DBT modes. In this part of the study, multiple phantom thicknesses were used to minimize the effect of breast thickness variation, which could be present in the clinical case. In our last approach, a rectangular phantom of 45 mm PMMA was used, and the AGD was estimated using an electronic dosemeter to provide a more robust dosimetric comparison of the dose.

### 2.1 Mechanical Imaging Sensors

**Fig. 1:**
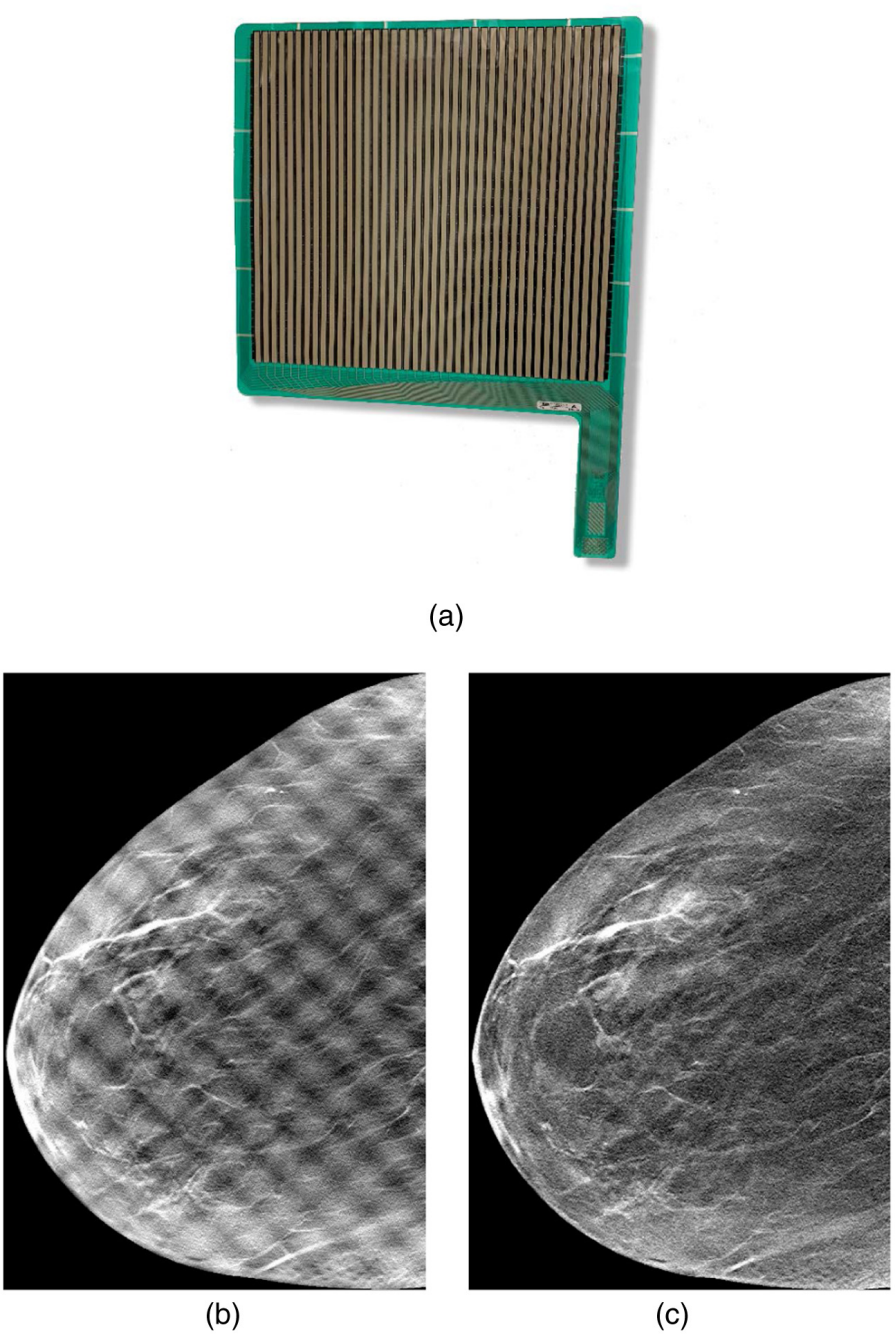
CONFORMAT 5350 pressure sensor from Tekscan that was used for MI measurements in this study. Examples of clinical reconstructed DBTMI images (b) before and (c) after post-processing to reduce sensor artifacts.

Our MI device was the CONFORMAT 5350 pressure sensor (Tekscan Inc., South Boston, Massachusetts, United States); see Fig. 1(a). The pressure grid consisted of multiple sensor elements (10.16  mm×10.16  mm) arranged in a matrix of 38×41 elements. Examples of clinical DBTMI images before and after post-processing to reduce sensor artifacts are shown in Figs. 1(b) and 1(c), respectively.

When used in our clinical study, the sensor was placed on the breast support. A radiographer was instructed to compress the breast in the same way as in clinical practice, and mechanical pressure was recorded during the whole compression procedure.

### 2.2 Estimation of AGD Increase based on Clinical Exposures

The clinical data used in this study originated from an ongoing clinical study of the simultaneous acquisition of DBT and MI conducted at the Unilabs Breast Center at Skåne University Hospital, Malmö.^24^ Women recalled from breast cancer screening for diagnostic work-ups were invited to participate. Approval for the study was obtained from the Swedish Ethical Review Authority (reference number: 2021-00606). During the recall visit, both oral and written information about the study were provided, and written consent was obtained from the participants. All participating women had had at least one DBT exam as part of their clinical diagnostic protocol. For the purpose of our study, one additional DBT acquisition view was performed with simultaneous MI acquisition (DBTMI) using AEC, as in the clinical acquisition. All study participants were imaged on a Mammomat Inspiration system (Siemens Healthineers, Forchheim, Germany). For each woman, the mammographic view [cranio-caudal, (CC) or medio-lateral oblique (MLO)] acquired for the study was chosen based on where the finding leading to recall was most visible in the DM screening examination. In this study, clinical data from 97 women were included in the analysis. Information on breast thickness and exposure corresponding to the central DBT projection was extracted from the DICOM headers—one extraction from the clinical DBT acquisition and one from the DBTMI acquisition. The breast thickness and exposure parameters were plotted as histograms for visual comparison between the two groups, and the overall increase in breast thickness and exposure between the clinical DBT and DBTMI groups was calculated and evaluated.

### 2.3 Estimation of AGD Increase Based on Phantom Measurements

**Fig. 2:**
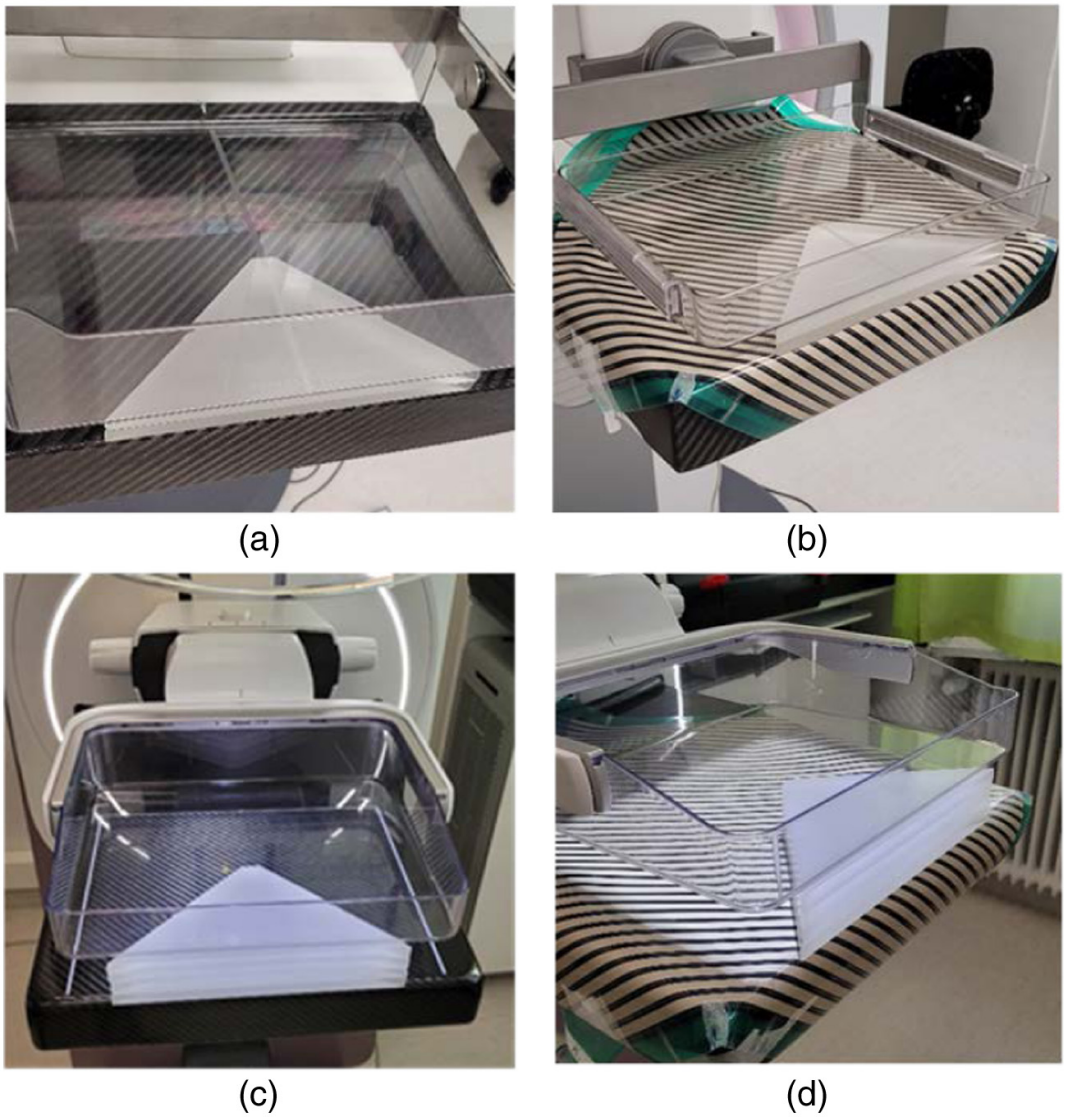
Setup for estimating AGD from DICOM headers using (a) and (b) 20 mm triangular PMMA blocks on the Siemens Inspiration system and (c) and (d) 40 mm triangular PMMA blocks on the GE Pristina system. In panels (b) and (d), the setup is shown with the MI sensor placed on the detector support.

In this part of the study, two clinically available imaging systems were used to expand our analysis and perform a phantom dose evaluation. These were the Mammomat Inspiration system as discussed above and the Senographe Pristina system (General Electric, Chicago, Illinois, United States). The acquisition parameters were automatically generated by the AEC settings for each of the systems, which were “OPDOSE” for the former system and “DOSE-” for the latter. These protocols are used clinically at our institution. Image acquisitions using various thicknesses of PMMA (20, 40, and 60 mm) for both DM and DBT modes in both imaging systems were performed and repeated with the MI sensor placed on the breast support (Fig. 2). Exposure and AGD information were extracted from DICOM headers of acquired phantom images.

### 2.4 Estimation of AGD Increase using an Electronic Dosemeter

Alongside the clinical exposures and estimated AGD to the phantom, determination of the AGD increase was also performed based on air kerma measurements using an electronic dosemeter (Raysafe X2 with a MAM sensor, Billdal, Sweden). The dosemeter was calibrated for photon energies typically used in breast imaging. Determination of the AGD, D, was conducted by following the procedure described in the European guidelines and Eq. (1), where the incident air kerma, K, is measured at the top of the phantom in contact with thee compression paddle.^29^ Conversion factors correspond to breast glandularity of 50%, g; various breast compositions, c; and differences depending on the selected target/filter combination, s.

D=K·g·c·s. (1)

For DBT, the AGD was estimated using Eq. (2). The incident air kerma, $K_{T}$, is measured at the 0° projection, and the tomofactor, T, is introduced to summarize the effect of various DBT projections that originate from different angles to the detector.^30^

$$D=K_{T}\cdot g\cdot c\cdot s\cdot T.$$ (2)

Dose estimations were conducted on the wide-angle Mammomat Inspiration system with an electronic dosemeter. According to the Dance standard breast model,^31^ a 45 mm rectangular PMMA phantom was used, and an air gap of 8 mm was included to reach a desired final breast thickness that mimicked the real breast composition.

**Fig. 3:**
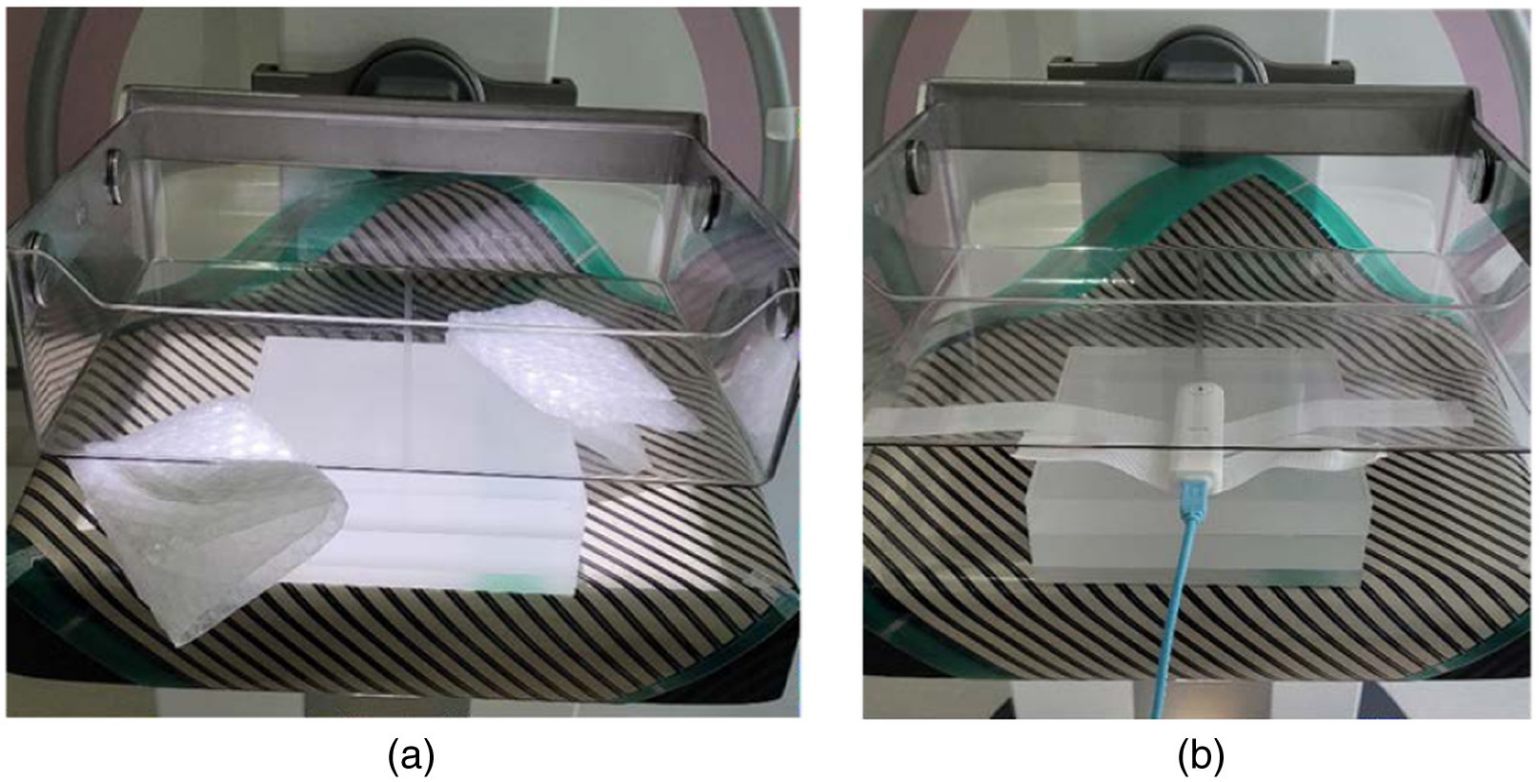
(a) Dose measurement setup using AEC settings. (b) The settings determined by the AEC were used in manual mode; the electronic dosemeter was used to measure the incident air kerma on the Siemens Inspiration system. In panels (a) and (b), the MI sensor was placed on the detector and below the PMMA blocks (similar to clinical acquisition).

Acquisitions were conducted using the AEC mode. Bubble wrap spacers were used to achieve an airgap of 8 mm and place the compression paddle at 53 mm from the breast support [Fig. 3(a)]. Each acquisition was repeated five times with maintained compression (the automatic release of the compression paddle was disabled).

Five additional acquisitions were then made with the electronic dosemeter positioned as described below using manual exposure settings with acquisition parameters as close as possible to the ones determined by the AEC from the first measurements. The reason for this setup was so the presence of the dosemeter did not interfere with the AEC settings. According to the European guidelines, the incident air kerma was measured using the electronic dosemeter centered with the sensitive area placed at 60 mm from the chest wall edge [Fig. 3(b)].^29^ To fit the electronic dosemeter between the phantom and the compression paddle, a breast thickness of 70 mm was set. The incident air kerma measured at 70 mm was recalculated to 53 mm using the inverse square law. Again, the automatic release of the compression paddle was disabled between measurements.

The procedure described above using the AEC mode and manual mode was repeated for the DM and DBT modes with and without the MI sensor placed on the breast support and below the PMMA phantom. In DM mode, the AGD values were calculated using Eq. (1), and the corresponding calculations in DBT mode were made using Eq. (2).

### 2.5 Statistical Analysis

Data were analyzed and visualized using MATLAB (version R2023a). A two-sided paired samples t-test was used to analyze differences in exposure and compressed breast thickness between the clinical DBT and the simultaneous DBTMI.

## 3 Results

### 3.1 Estimation of AGD Increase Based on Clinical Exposures

Ninety-eight women participated in this study. Images from one woman were excluded due to technical reasons (corrupt MI files). Image acquisition information was extracted from the DICOM headers of the 97 recalled women included in the analysis. All data were anonymized. Out of the 97 recalled women, 91 were examined with DBTMI using CC view and 6 using MLO view.

**Fig. 4:**
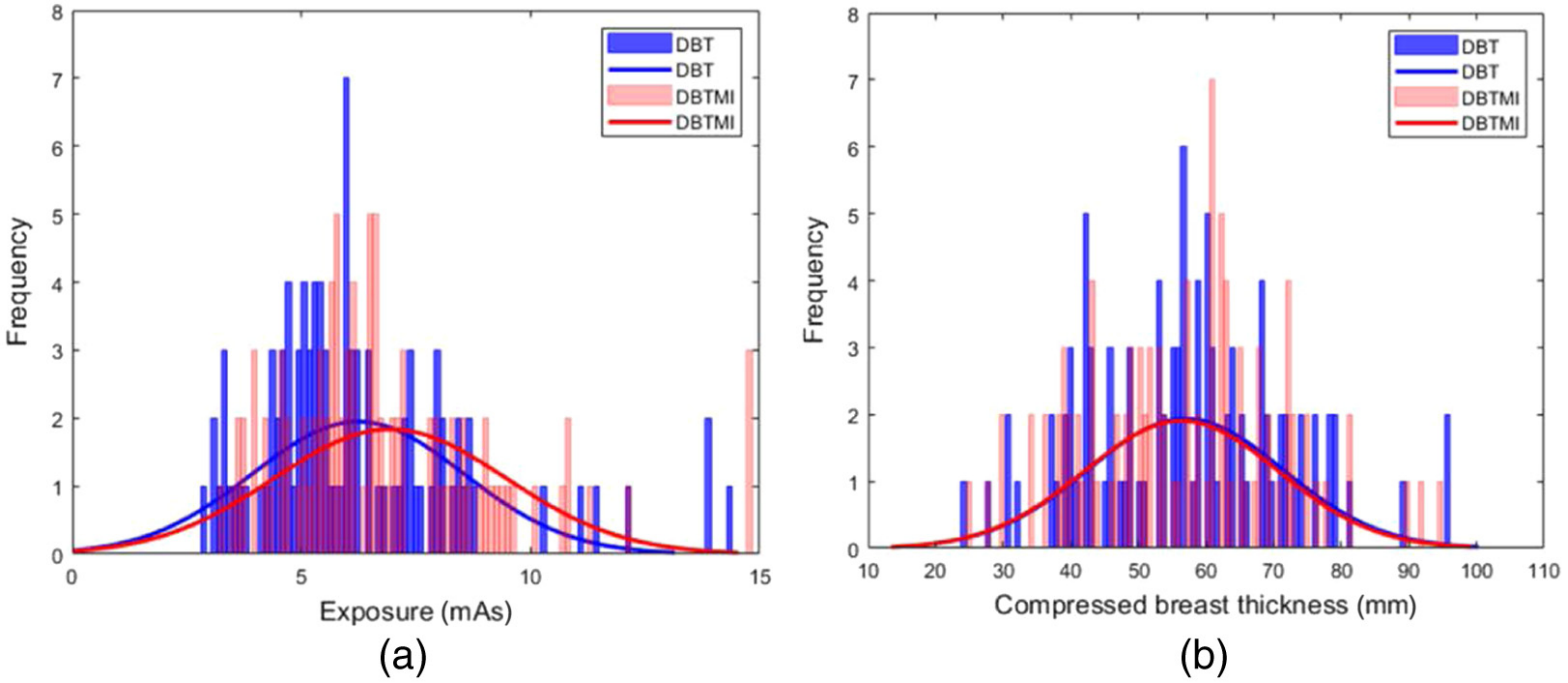
Histogram of (a) exposure parameters and (b) compressed breast thickness observed for acquisitions made in clinical DBT and DBTMI mode, respectively.

Figure 4 shows the histograms of the exposure values and compressed breast thicknesses in clinical DBT and simultaneous DBTMI among the 97 women analyzed in this study; the mean and standard deviation are compared in Table 1. The relative increase in exposure between DBT and DBTMI was 11.9%±10.4.

**Table 1:** Mean and standard deviation of the exposure values and compressed breast thickness for clinical DBT exams in this study.

	Exposure (mAs)	Compressed breast thickness (mm)
DBT	6.2±2.3	57.0±14.4
DBTMI	6.9±2.5	56.4±14.3
*p*-value	<0.0001	0.0485
95% CI	(−0.8379, −0.5716)	(0.0037, 1.1303)

The histogram for simultaneous DBTMI in Fig. 4(a) is shifted toward higher exposure values compared with clinical DBT, indicating a significant difference between the two groups. In Fig. 4(b), the corresponding histogram of the compressed breast thickness is visualized, indicating no significant difference between the two groups at the population level.

The distribution of changes in breast thickness between clinical DBT and simultaneous DBTMI shows that 30.9% of the women had an increase in breast thickness, 50.5% had a decrease in breast thickness, and the remaining 18.6% showed no change in breast thickness.

### 3.2 Estimation of AGD Increase Based on Phantom Measurements

**Fig. 5:**
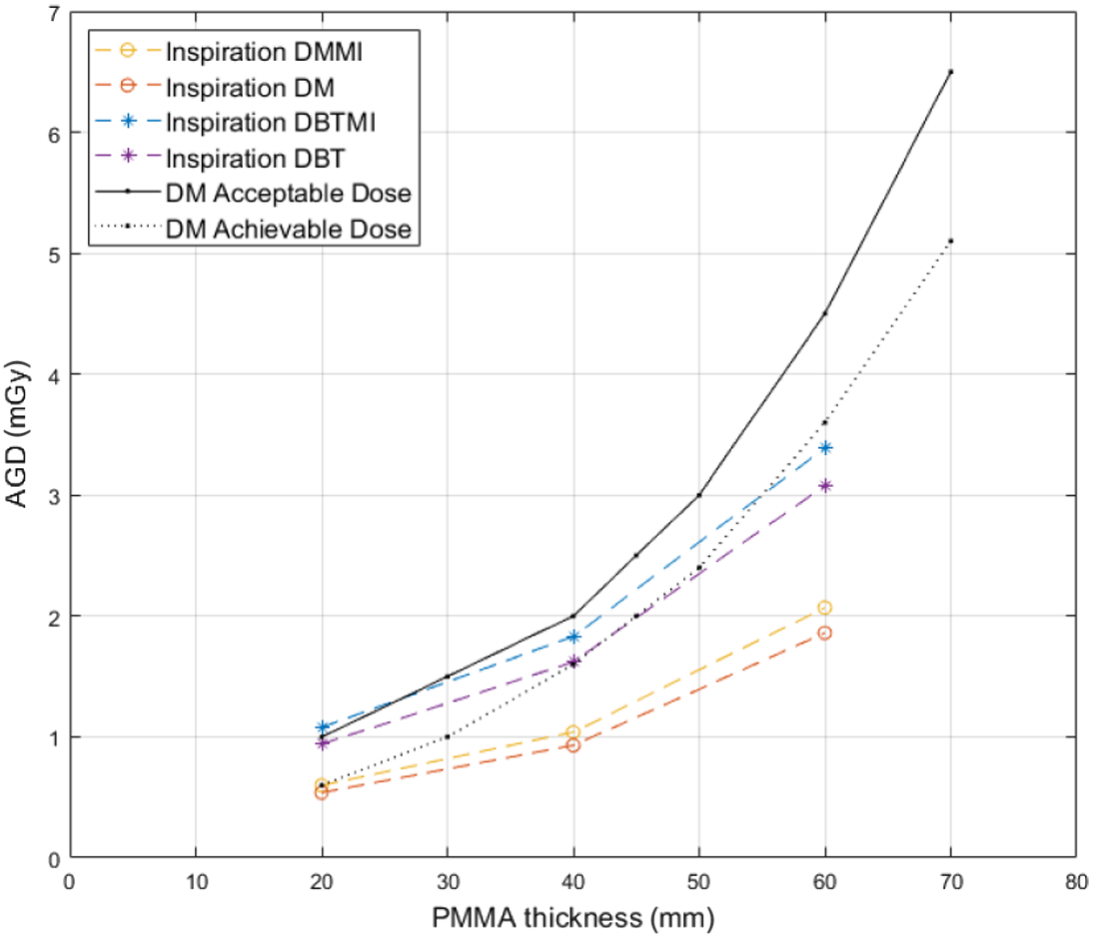
AGD as a function of PMMA thickness in DM and DBT with and without the sensor present for the Mammomat Inspiration mammography system. Acceptable and achievable DM dose levels as recommended by the European protocol for quality control in mammography^32^ are indicated.

The estimated AGD reported by the imaging system when placing the MI sensor on the breast support versus not using the MI sensor during DM and DBT acquisition is presented in Fig. 5 for the Mammomat Inspiration system. PMMA thicknesses of 20, 40, and 60 mm were used.

**Fig. 6:**
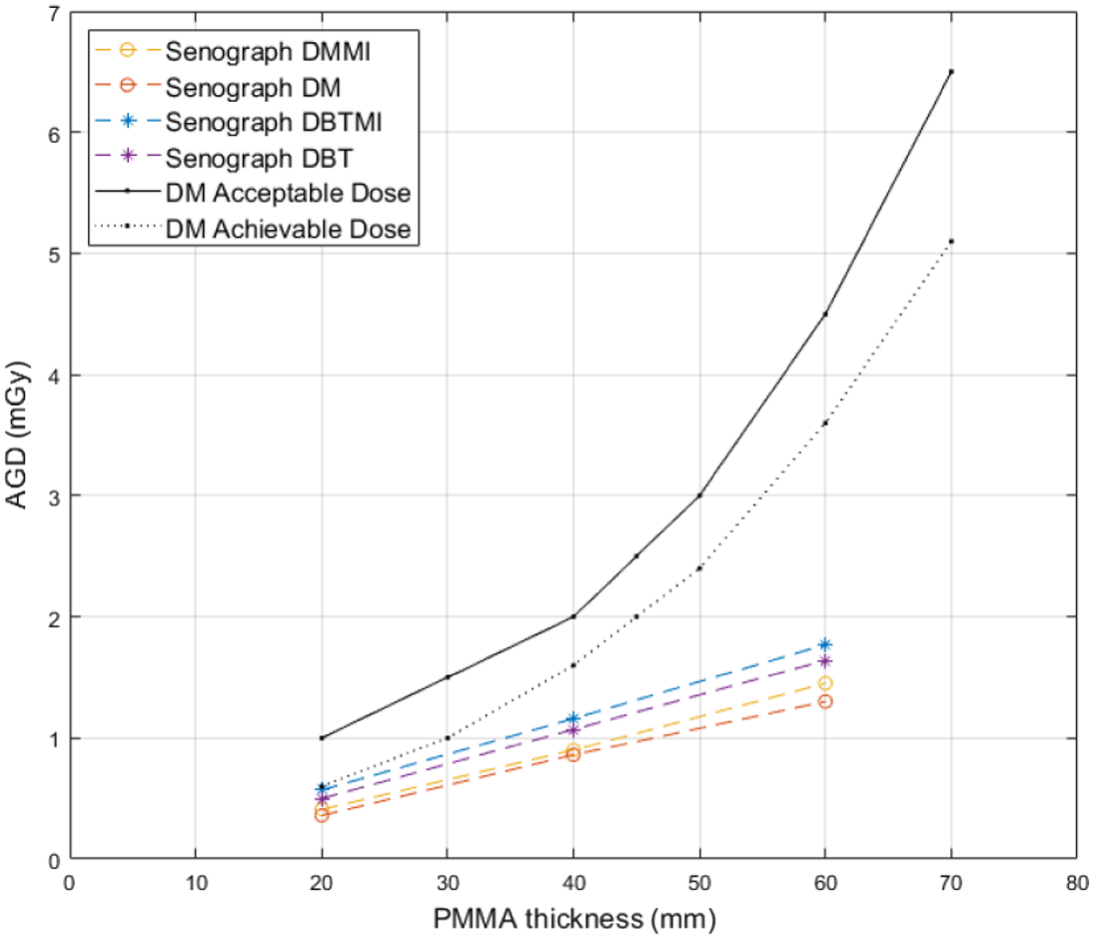
AGD as a function of PMMA thickness in DM and DBT with and without the sensor present for the Senographe Pristina mammography system. Acceptable and achievable DM dose levels as recommended by the European protocol for quality control in mammography^32^ are indicated.

Figure 6 represents the AGD estimated using the same approach, with the GE Senograph Pristina system.

Acceptable and achievable DM dose levels, recommended in Europe^32^ are also shown in Figs. 5 and 6. No significant difference in dose increase between the two systems could be observed. The increase in dose averaged over different PMMA thicknesses for the Inspiration system was 11.4%±0.4 (DM) and 12.6%±2.4 (DBT), and the increase in dose for the Senographe system was 10.0%±4.8 (DM) and 10.1%±3.4 (DBT). On average for both imaging systems, the relative increase in dose with the sensor present for DM acquisitions was 10.7%±3.1. For DBT, the corresponding relative increase in dose was 11.4%±3.0.

### 3.3 Estimation of AGD Increase Using an Electronic Dosemeter

The AGD and standard deviation estimated using the electronic dosemeter with and without the MI sensor in DM and DBT modes are presented in Table 2. The relative dose increase for DM mode was 11.2%, and it was 12.2% for DBT. The average difference in dose over all dose estimation methods was 11.5%±3.3.

**Table 2:** Estimated AGD values from measurements using the electronic dosemeter.

	AGD (mGy)	AGD increase (%)
DM	1.12±0.001	11.2
DMMI	1.25±0.001	
DBT	1.91±0.005	12.2
DBTMI	2.14±0.007	

## 4 Discussion

This paper analyzes increases in the radiation dose to the breast when performing MI in conjunction with acquiring an X-ray image of the breast. The dose increase is caused by the presence of the MI sensor on the breast support. Previously, alternative positioning of the MI sensor has been investigated. In a study of MI as adjunct to DM in two separate acquisitions, the sensor was placed on the compression plate.^20^ Such positioning did not increase the dose to the breast. In simultaneous DBTMI, however, the sensor is positioned preferably on the breast support, to facilitate the suppression of sensor artifacts in DBT projections.^22^ Positioning the sensor on the compression plate would result in varying artifacts, due to geometric magnification. The artifact magnification would vary with breast thickness and prevent artifact suppression.

Our results are based on an analysis of clinical exposures and phantom measurements. When combining the results from both DM and DBT acquisitions, our measurements suggest that the simultaneous use of MI and breast radiography increases the AGD to women by on average 11.5%. This increase in AGD is justified because of the valuable additional information that is provided through MI. The absolute AGD during simultaneous DM/DBT and MI was 1.25 mGy in DM mode and 2.14 mGy in DBT mode. The acceptable dose limit according to the European Commission for a 53 mm breast thickness, or 45 mm PMMA, is 2.5 mGy, and thus, both resulting doses are below the acceptable dose limit. Hence, our results suggest that the increase in AGD would not be a limitation for MI implemented in the clinical workflow.

Our first approach to estimate increased AGD when performing simultaneous DBTMI showed a statistically significant difference in dose between DBT and DBTMI. One potential source of uncertainty in dose estimation when using DBTMI simultaneously could be the fact that the MI sensor may affect the breast compression made by the radiographer. Our results showed that the breast compression was roughly the same with and without the sensor present. However, when the fractions were analyzed, it turned out that 18.6% of women had the same compressed breast thickness, whereas 50.5% had a decrease in breast thickness when performing DBTMI compared with DBT. This could be the result of clinically observed variation in breast compression.^33^ Our second and third approaches to estimate increased AGD in DMMI and DBTMI were conducted using solid PMMA phantoms, eliminating the breast thickness variation with and without the MI sensor, and those measurements showed results similar to the clinical measurements.

The estimations of increased AGD in Sec. 2.2 have used the AGD values reported by the imaging system. The reported AGD was based on assumptions of a standard breast and did not include corrections for any metallic elements, such as those existing in our MI sensor. Hence, the dose estimation reported in the DICOM header might lack proper validation for DBTMI acquisition. This could be investigated further using multiple point dosemeters, such as thermoluminescent dosemeters. One other option could be to use optically stimulated luminescent dosemeters made out of NaCl pellets, which is an affordable method and especially suitable due to the possibility of multiple point measurements. The use of optically stimulated luminescent dosemeters with NaCl pellets is a novel technique within the clinical field, but it has shown promising results in initial measurements.^34^

It can be observed that the increase in AGD was within the same order of magnitude as our previously reported results in the comparison among clinical exposures in Sec. 3.1 and our phantom measurements of increased AGD in Sec. 3.2. Our results in Sec. 3.1, which showed an increase in exposure of 11.9% compared with DBT when performing a simultaneous DBTMI, were expected. They were within the same order of magnitude as our previous preliminary reported increased dose.^28^

## 5 Conclusions

We estimated the cost, in terms of radiation dose, of simultaneous breast radiography and MI due to the presence of an MI sensor with X-ray attenuating metallic elements. The results observed are in agreement with our preliminary study of doses in DBTMI and DBT in women recalled from screening. In our study, an overall dose increase of 11.5% was suggested. Based on the clinical guidelines for radiation doses in mammography, the observed AGD values should not limit the potential use of DBTMI as an additional modality of choice in breast cancer screening.

## Data Availability

All images are stored locally in easily read DICOM format. The dosimetric data are recorded in Microsoft Excel worksheets. The archive of all data is available by request from the corresponding author.
